# Lignin-Based Polyurethanes from the Blocked Isocyanate Approach: Synthesis and Characterization

**DOI:** 10.1021/acsomega.3c03422

**Published:** 2023-07-18

**Authors:** Leonardo D. Antonino, Ivan Sumerskii, Antje Potthast, Thomas Rosenau, Maria Isabel Felisberti, Demetrio J. dos Santos

**Affiliations:** † Nanoscience and Advanced Materials Graduate Program (PPG-nano), Federal University of ABC (UFABC), Santo André 09210-580, Brazil; ‡ Department of Chemistry, Division of Chemistry of Renewable Resources, University of Natural Resources and Life Sciences Vienna (BOKU), Konrad-Lorenz-Strasse 24, 3430 Tulln an der Donau, Austria; § Institute of Chemistry, University of Campinas (UNICAMP), P.O. Box 6154, Campinas 13083-970, Brazil; ∥ Center of Engineering, Modeling and Applied Social Sciences, Federal University of ABC (UFABC), Santo André 09210-580, Brazil

## Abstract

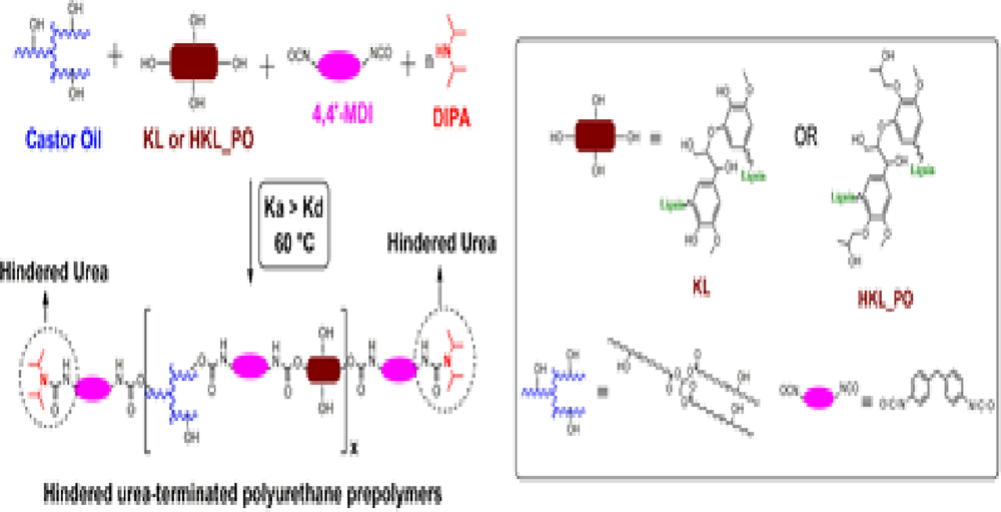

Lignin, the world’s second most abundant biopolymer, has been investigated as a precursor of polyurethanes due to its high availability and large amount of hydroxyls present in its structure. Lignin-based polyurethanes (LPUs) are usually synthesized from the reaction between lignin, previously modified or not, and diisocyanates. In the present work, LPUs were prepared, for the first time, using the blocked isocyanate approach. For that, unmodified and hydroxypropylated Kraft lignins were reacted with 4,4′-methylene diphenyl diisocyanate in the presence of diisopropylamine (blocking agent). Castor oil was employed as a second polyol. The chemical modification was confirmed by ^31^P nuclear magnetic resonance (^31^P NMR) analysis, and the structure of both lignins was elucidated by a bidimensional NMR technique. The LPUs’ prepolymerization kinetics was investigated by temperature-modulated optical refractometry and Fourier-transform infrared spectroscopy. The positive effect of hydroxypropylation on the reactivity of the Kraft lignin was verified. The structure of LPU prepolymers was accessed by bidimensional NMR. The formation of hindered urea-terminated LPU prepolymers was confirmed. From the results, the feasibility of the blocked isocyanate approach to obtain LPUs was proven. Lastly, single-lap shear tests were performed and revealed the potential of LPU prepolymers as monocomponent adhesives.

## Introduction

Polyurethanes (PUs) are a versatile class of polymers conventionally synthesized through reactions between polyols and diisocyanates. These materials have an outstanding potential for use in a wide range of applications, such as coating, adhesives, sealants, flexible and rigid foams for thermal insulation, and biomedical devices.^1^ Industrially, both PU precursors are usually derived from petroleum.^2^ However, due to environmental concerns and the imminent depletion of fossil resources, the development of biobased PUs has gained notoriety during the past decades.^3−5^ Among several candidates, lignin, a natural polymer found in the biomass, has been standing out as possible starting component for biobased PUs due to its large natural and industrial availability as a byproduct obtained from the pulp and paper industry, in addition to its high content of hydroxyls groups and high mechanical strength.^6^

Lignin is the second most abundant biopolymer on the planet and the most abundant aromatic one.^7^ It is found in plant cells together with cellulose and hemicellulose. Its aromatic structure is composed of three basic phenylpropane units, guaiacyl (G-unit), syringyl (S-unit), and *p*-hydroxyphenyl (H-unit), which are derived from coniferyl, sinapyl, and *p*-coumaryl alcohol, respectively.^8^ These units are linked by carbon–carbon (β–5, 5–5, β–1, and β–β) and carbon–oxygen linkages (β–O–4, α–O–4, and 4–O–5), forming an amorphous and complex three-dimensional structure with a variety of functional groups, such as carbonyl, methoxyl, and aliphatic and phenolic hydroxyl groups.^9,10^ Among the functional groups, the hydroxyls are the most abundant and most reactive and therefore the preferred target for reactions to develop lignin-based materials, such as PUs. Furthermore, the abundant aromatic moieties impart high stiffness to lignin.^11^ Over the past decades, different types of lignin-based PUs (LPUs), such as foams,^12,13^ elastomers,^14^ coatings,^15−17^ and adhesives,^18−20^ have been reported in the literature.

Despite its great potential, lignin has some limitations in the context of PU synthesis. First, its use alone, as the only polyol in PU formulations, is disadvantageous because it results in highly brittle materials.^11^ For that reason, lignin is frequently blended with other polyols, such as polyethylene glycol (PEG),^21^ a conventional petroleum-based polyol, and, in a sustainable approach, vegetable oils, such as castor oil for example.^22,23^ This second polyol acts as the soft segment in the lignin-based PU formulation. Even with the addition of the second polyol, lignin can only be incorporated up to 30 wt % without deterioration of the PU’s mechanical properties.^24,25^ Another drawback is the low reactivity of the lignins’ hydroxyl groups against isocyanates, especially aromatic ones, due to steric hindrance.^26^ To overcome this limitation, several methods of lignin derivatization have been proposed. Among these approaches, the hydroxyalkylation with propylene oxide, also known as hydroxypropylation, is the most employed one.^27^

Hydroxypropylation modifies the structure of lignin by adding aliphatic chains with secondary hydroxyls (hydroxypropyl units).^28^ Conventionally, this modification route is carried out at a high pressure and temperature. However, more recent works reported successful hydroxypropylation at room temperature and atmospheric pressure.^29,30^ Under this reaction condition, the propylene oxide’s homopolymerization is avoided, and only the aromatic lignin hydroxyls are derivatized, resulting in a powder lignin with high reactivity against isocyanate groups and, consequently, in a PU with better properties.^30^ Hydroxypropylated lignins have been employed in formulations of biobased PU adhesives^22,31−33^ and foams.^27,34,35^

Despite the considerable number of works investigating the synthesis of LPU, there are no studies reported in the literature that employ blocked isocyanate chemistry. Here, this work sets in novel LPUs that were developed from pristine and chemically modified technical lignins, following the blocked isocyanate approach, in which isocyanate groups react initially with a compound called the blocking agent. This leads to the formation of a chemically stable functional group, which, at first, prevents the reaction with polyol hydroxyls and, consequently, the formation of urethane linkages. The formation of urethane linkages, i.e., the actual crosslinking reaction, only occurs above a certain temperature, known as the deblocking temperature, at which the dissociation of the previously formed protecting group is effected, resulting in isocyanate “deblocking”.^36^ Blocking agents are compounds with active hydrogens, such as phenols, alcohols, amines, amides, oximes, imides, and imidazoles.^37^ Although not being the most usual approach, the chemistry of blocked isocyanates is widely used to obtain PU adhesives and has some advantages over traditional synthesis methods, such as less sensitivity to moisture, less toxicity due to the low concentration of free isocyanates, and higher stability during storage.^38^ Furthermore, this methodology can also be used to control the molar mass, polydispersity, and molecular architecture of PU.^39^ Polo Fonseca and Felisberti^40^ developed a synthetic route based on the equilibrium dissociation of sterically hindered ureas called dynamic urea bond-mediated polymerization (DUBMP). The blocked isocyanate, *N*,*N*-diisopropyl urea, is the product of the reaction between an isocyanate and a blocking agent, in this case *N*,*N*-diisopropylamine, a secondary amine. The PU was synthesized from a mixture of isophorone diisocyanate (IPDI), 1,4-butanediol, and the blocking agent, the amine reacting much faster with the isocyanate than the alcohol. At 110 °C, the deblocking temperature, the urea intermediates become unstable and decompose. The resulting isocyanates, formed in situ, react with the hydroxyl functions to stable urethane moieties. The authors obtained PU with a low molar mass (*M*_W_ < 5000 g·mol^–1^) and polydispersity (<1.3). In another work,^41^ the same authors replaced 1,4-butanediol, a bivalent alcohol, by a mixture of polyols, poly(ethylene glycol) and polycaprolactone triol, with a functionality higher than 2, similar to lignin. The synthesized PU showed a high molar mass, dispersity, and degree of branching.

In this work, novel LPUs were prepared either from unmodified Kraft lignin or from hydroxypropylated lignin using blocked isocyanate chemistry. The synthesis methodology was based on the DUBMP approach. In addition to lignin, castor oil and methylene diphenyl diisocyanate (MDI) were used in the PU formulations. The chemical modification of lignin was confirmed by ^31^P nuclear magnetic resonance (^31^P NMR) spectroscopy. 2D NMR (HSQC) analysis was carried out for structural elucidation of unmodified and hydroxypropylated lignin. To the best of our knowledge, HSQC NMR was employed for the first time to an in-depth investigation of the LPU’s molecular structure. Additionally, the prepolymerization process was studied using temperature-modulated optical refractometry (TMOR) and Fourier-transform infrared (FTIR) spectroscopy. The results show that the blocked isocyanate approach is feasible for the synthesis of LPU. The synthesized prepolymers have a good potential to be applied as heat activated adhesives or coatings or similar high-value materials. Finally, the results add another facet to the already versatile application of 2D NMR techniques in lignin chemistry, namely, the characterization of LPUs.

## Materials and Methods

### Materials

Kraft lignin (KL) from eucalyptus hardwood was kindly supplied by Suzano S.A. (Brazil). Castor oil was purchased from Azevedo (Brazil) with a hydroxyl value of 159 mg KOH·g^–1^. Propylene oxide (PO), 4,4′-methylene diphenyl diisocyanate (4,4′-MDI) with 32.9% NCO groups (according to the supplier), *N*,*N*-diisopropylamine (DIPA), deuterated dimethyl sulfoxide-*d*_6_ (DMSO-*d*_6_), deuterated chloroform (CDCl_3_), pyridine, and hydrochloric acid (HCl) were purchased from Sigma Aldrich in the highest purity grade available and used as received. NaOH was supplied by Nox Lab Solutions (Brazil). The ^31^P NMR derivatization agent 2-chloro-4,4,5,5-tetramethyl-1,3,2-dioxaphospholane was obtained from ChiroBlock GmbH (Germany) in the highest purity grade available. The NMR relaxation agent chromium acetylacetonate [Cr(acac)_3_] (>95%) was provided by Fluka. The internal standard (IS) for ^31^P NMR, *N*-hydroxy-5-norbornene-2,3-dicarboxylic acid imide (>99%, e-HNDI), was supplied by TCI GmbH (Germany).

### Lignin Hydroxypropylation

KL was dried for 24 h in an oven with air circulation at 60 °C. The hydroxypropylation protocol was based on García et al.^29^ Briefly, 6 g of KL was solubilized in aqueous NaOH solution (30 mL, 2.5 M). After total homogenization, 7.5 mL of propylene oxide (PO:OH_phenolic_ = 4.0) was added dropwise to the solution. The mixture was kept at room temperature for 1 h. During this period, the pH was controlled by the addition of HCl and kept between 10 and 11. The mixture was cooled to room temperature and allowed to rest for 24 h. The hydroxypropylated lignin (HKL_PO) was precipitated into aqueous HCl solution (pH = 2), vacuum-filtrated, and washed five times with deionized water. The brown powder obtained was dried in an oven at 60 °C.

### Preparation of Hindered Urea-Terminated Polyurethane Prepolymers

The preparation of PU prepolymers, temporarily blocked isocyanates with thermally cleavable protecting groups, was based on a protocol by Polo Fonseca and Felisberti.^40^ Renewable polyols were obtained by blending either KL or HKL_PO with castor oil (castor oil:lignin mass ratio = 70:30). The raw materials were manually mixed for 2 min to achieve the desired homogeneity. Thereafter, DIPA, the blocking agent, was added to the polyol blend at an OH:DIPA molar ratio of 1:0.5. The mixture was mixed for another 2 min. MDI was added to the mixture (NCO:OH molar ratio = 1:1). The systems were manually mixed for 3 min to ensure complete homogenization. Polymerization was carried out at 60 °C for 3 h. The blocked prepolymers containing KL or HKL_PO were labeled as BPUP_30KL and BPUP_30HKL_PO, respectively.

### ^31^P Nuclear Magnetic Resonance Spectroscopy (^31^P NMR)

^31^P NMR quantitative analysis was carried out to identify changes in the OH amount of KL after hydroxypropylation. ^31^P NMR was performed on a Bruker Avance II 400 MHz spectrometer (Bruker, Germany) equipped with a 5 mm broadband observe probe head, with the *z*-gradient at r.t. (standard Bruker pulse programs) according to Korntner et al.^42^ First, 25 mg of lignin (KL or HKL_PO) was completely dissolved in 700 μL of a 1:1.6 (v/v) mixture of CDCl_3_ and pyridine (nondeuterated) by shaking only at room temperature. Afterward, 200 μL of a stock solution containing the IS (0.02 mmol·mL^–1^) and the NMR relaxation agent, chromium acetylacetonate [Cr(acac)_3_; 5 mg·mL^–1^], was added. After thorough mixing, 100 μL of the phosphitylation reagent (2-chloro-4,4,5,5-tetramethyl-1,3,2-dioxaphospholane) was injected through a septum into the vial to avoid any contact of the reagent with moisture. The samples were shaken for 1 h at room temperature and then transferred into NMR tubes. Acquisition parameters: 25 °C, 160 scans, and a 14 s delay between pulses. The calculation of OH groups was based on the integration of the following spectral regions: OH_aliphatic_ (150.0–144.6 ppm), 5-substituted units (144.6–140.4 ppm), guaiacyl-OH (140.4–138.3 ppm), *p*-hydroxyphenyl-OH (138.3–137.0 ppm), and carboxylic acids (136.0–133.6 ppm). Spectral processing and integration were done with Bruker TopSpin version 4.1.4.

### Two-Dimensional Nuclear Magnetic Resonance Spectroscopy (HSQC)

The 2D HSQC NMR technique was used to elucidate the chemical structure of unmodified and hydroxypropylated lignin, as well as PU prepolymers. Spectra were acquired at 25 °C on a Bruker Avance II 400 MHz spectrometer (Bruker, Germany). The sample (40 mg) was dissolved in 600 μL of DMSO-*d*_6_ and then transferred into an NMR tube. A spectral width of 11 ppm was chosen in the ^1^H domain and 160 ppm in the ^13^C domain. Data were acquired in an 800 × 256 *k*-point data matrix with a scan number of 80 and a relaxation delay of 0.5 s. Data processing was carried out using Bruker TopSpin 4.1.4. The assignment of the signals was supported by the nmrdb.org tool.

### Fourier-Transform Infrared Spectroscopy (FTIR)

Fourier-transform infrared spectroscopy in the attenuated total reflectance mode (FTIR-ATR) was performed on a Spectrum Two instrument (PerkinElmer, USA) to investigate the evolution of the prepolymerization process of polyurethanes. Spectra of aliquots extracted in three different times (0, 1.5, and 3 h) were recorded between 3800 and 800 cm^–1^ with 32 scans and a resolution of 4 cm^–1^ at room temperature in an air atmosphere.

### Temperature-Modulated Optical Refractometry (TMOR)

TMOR is a novel dilatometry technique based on optical refractometry. Due to its recent development, we included that approach in more detail in the discussion. A brief theoretical background can be found in the Supporting Information. For a more detailed explanation concerning the physical background and theory, the reader is referred to original works.^43−45^

TMOR analysis was conducted on a thermo-optical oscillating refraction analyzer TORC 5000 (Anton Paar, Brazil), working at an absolute refractive index accuracy of ca. 10^–6^ and prism temperature accuracy of 10^–2^ °C. The homogeneous PU reactive mixtures containing the blocking agent DIPA (approx. 2 mL) were poured into the TMOR cavity. The prepolymerization process was monitored at an average temperature of 60 °C with a modulation period of 60 s and a temperature amplitude of 0.5 °C for 3 h. The evolution of the mean refractive index (*N*_Mean_) over this time was recorded and used as a basis to investigate the kinetics of this prepolymerization process.

### Single-Lap Shear Test

The applicability of the synthesized prepolymers as adhesives was evaluated by single-lap shear tests. The adhesive tests were performed in steel substrates, which consisted of two rectangular plates with dimensions of 100 mm × 25 mm × 1.5 mm. The overlap length for all samples was 12.7 mm. Three specimens of each material were tested. The adhesive was applied at both plates of each material, which were kept together at constant pressure using grips during the curing (150 °C for 6 h). An Instron 3369 universal testing machine was used for the tests, which were carried out at a speed rate of 2 mm·min^–1^ for all samples.

## Results and Discussion

### Lignin Characterization

#### ^31^P Nuclear Magnetic Resonance Spectroscopy (^31^P NMR)

The ^31^P NMR spectra of KL and HKL_PO are shown in Figures S1 and S2 (Supporting Information). The hydroxyl contents of KL and HKL_PO obtained by ^31^P NMR analysis are presented in Table 1. Almost 80% of the original KL’s phenolic OHs were converted to aliphatic ones after the reaction with PO. According to García et al.^29^ and Sadeghifar et al.,^30^ under the reaction conditions employed, the hydroxypropylation is quite chemoselective with only phenolic OHs being derivatized, but not the aliphatic ones. The results strongly support the successful hydroxypropylation of KL. Furthermore, HKL_PO showed a total OH content of 5.27 mmol·g^–1^ (excluding carboxylic OH), lower than KL (6.06 mmol·g^–1^). This apparent reduction is probably associated with the molar mass increase associated with the attachment of PO units in the lignin structure and some crosslinking.^46,47^ The small decrease in the carboxyl content verified for HKL_PO supports this hypothesis since this functional group theoretically should not react with PO considering the reaction conditions.

**Table 1:** Hydroxyl Group Content of Lignin Samples Obtained by ^31^P NMR Analysis

		OH_phenolic_ (mmol·g^–1^)						
lignin sample	OH_aliphatic_ (mmol·g^–1^)	syringyl	condensed guaiacyl	noncondensed guaiacyl	*p*-OH-phenyl	total	∑OH (mmol·g^–1^)	carboxyl (mmol·g^–1^)
KL	1.54	2.07	1.27	1.03	0.15	4.52	6.06	0.34
HKL_PO	4.31	0.33	0.37	0.22	0.04	0.96	5.27	0.29

#### Two-Dimensional Nuclear Magnetic Resonance Spectroscopy (HSQC)

**Chart 1:**
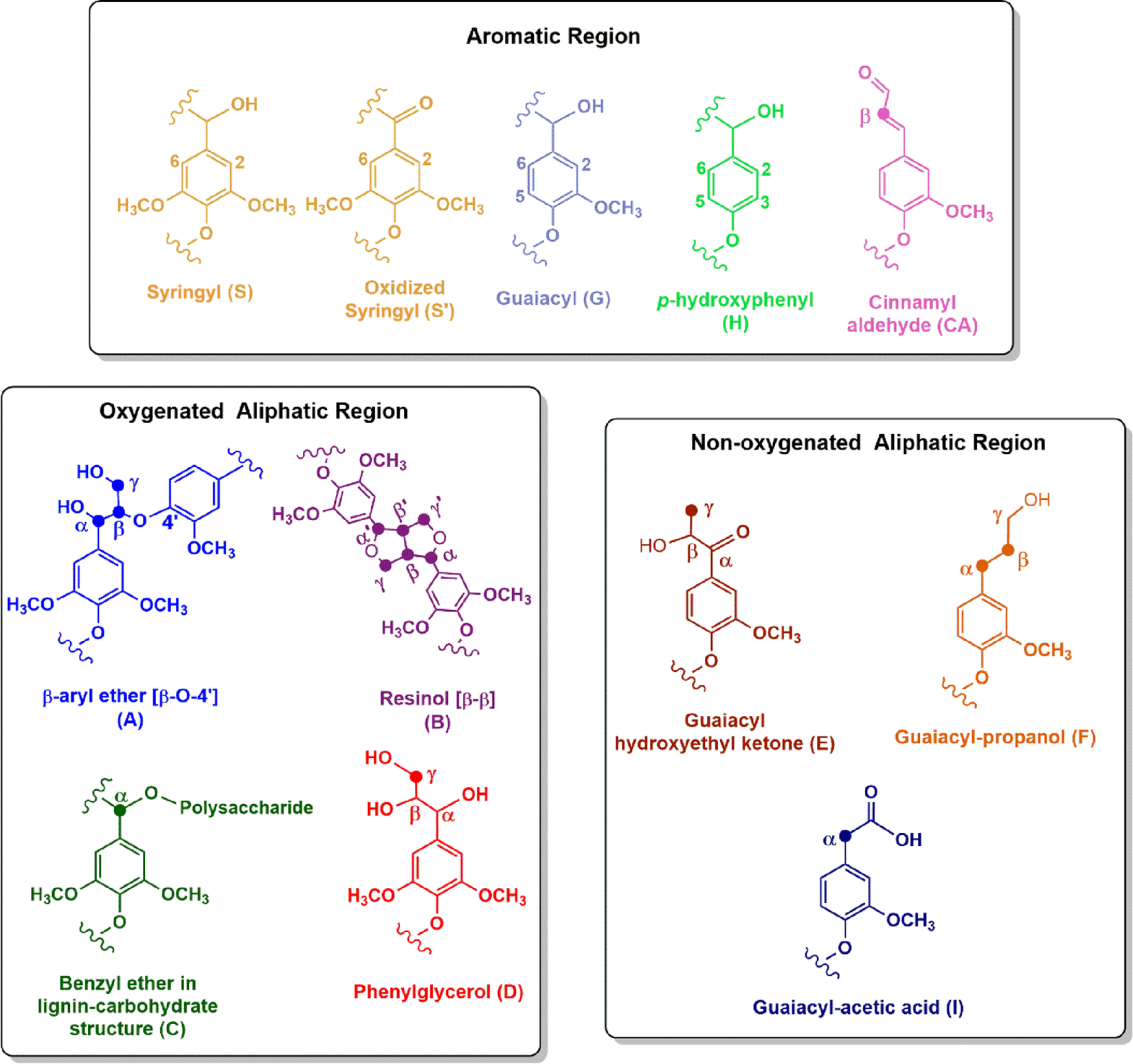
The Main KL Aromatic Structures and Side Chain Structures Identified, Separated by the Spectral Region

**Figure 1:**
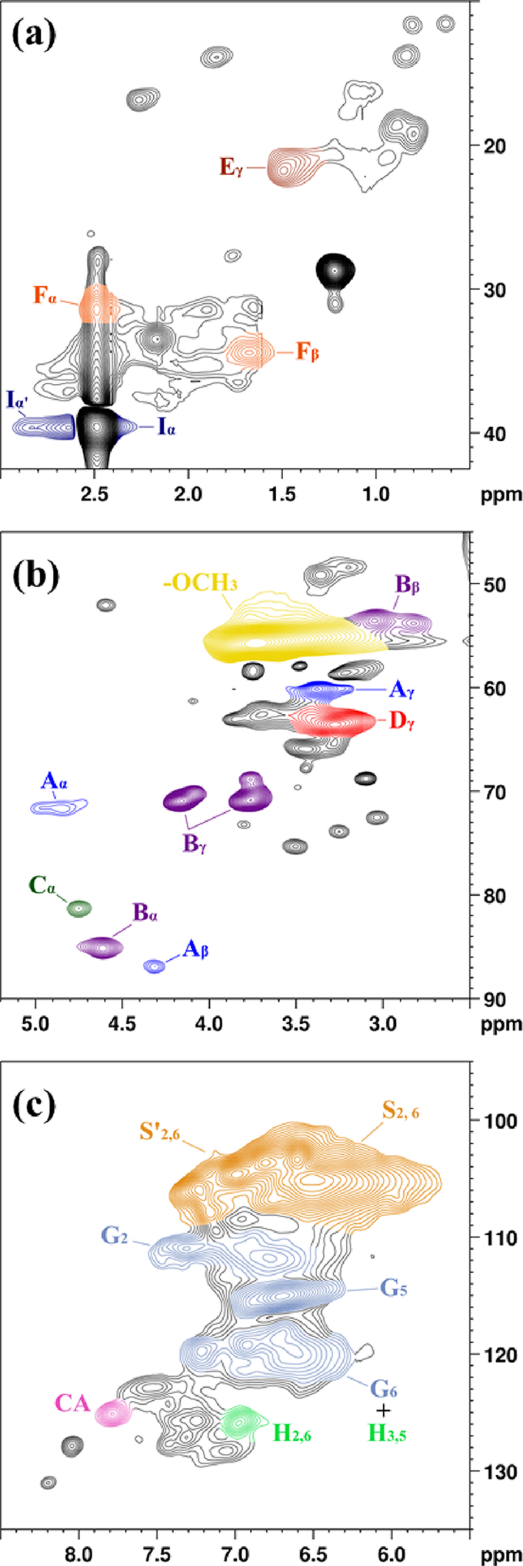
HSQC spectrum of Kraft lignin (KL) and assignments of selected structural features (see Chart 1): (a) aromatic region, (b) oxygenated aliphatic region, and (c) nonoxygenated aliphatic region.

The detailed chemical structure of KL and HKL_PO was investigated using the two-dimensional HSQC NMR technique. The HSQC spectrum of KL was divided into three regions: aromatic, oxygenated aliphatic, and nonoxygenated aliphatic (Figure 1). From the spectrum, signals for the main structural characteristics of lignins, including the basic composition, i.e., syringyl (S), guaiacyl (G), and *p*-hydroxyphenyl (H) units, and various lignins’ typical substructures linked by ether and carbon–carbon bonds can be observed. All the assigned structures are depicted in Chart 1. In the nonoxygenated aliphatic region (Figure 1a), substructures linked by carbon–carbon bonds, such as guaiacyl hydroxyethyl ketone (E, δ_C_/δ_H_ 22/1.5), guaiacyl-propanol (F, δ_C_/δ_H_ α: 32/2.5, β: 35/1.7), and guaiacyl-acetic acid (I, δ_C_/δ_H_ 39/2.35 and 39/2.65), were verified.^48^ The presence of guaiacyl-acetic acid structure supports the ^31^P NMR result (Table 1) since it confirms the presence of carboxylic hydroxyls. In the oxygenated aliphatic region (Figure 1b), lignins’ characteristic structures, such as β-O-4′aryl ethers (A, δ_C_/δ_H_ α: 72/4.9, β: 87/4.35, γ: 60/3.4), resinols (B, δ_C_/δ_H_ α: 84/4.65, β: 54/2.8 to 3.2, γ: 70/4.2 and 72/3.8), and phenylglycerol (D, δ_C_/δ_H_ 63/3.1 to 3.5), were identified.^49,50^ A signal related to benzyl ethers in lignin-carbohydrate (C, δ_C_/δ_H_ 81/4.75) structures was also observed, indicating the presence of linked carbohydrate traces in the KL structure. Although not identified in the present spectrum, phenylcoumaran (β-5′ linkage) structures are usually found in Kraft lignin, but in lower concentrations.^48,51^ Methoxyl groups (OMe, δ_C_/δ_H_ 52 to 57/3.1 to 4.0), lignin’s most typical functional groups, were also identified in this spectrum region. Finally, in the aromatic region (Figure 1c), signals associated with S (δ_C_/δ_H_ 101 to 109/5.9 to 7.4), G (δ_C_/δ_H_ 110 to 122/6.5 to 7.0), and H (δ_C_/δ_H_ 127/7.0) units were verified.^49,51^ The signal intensities suggested that KL is mostly composed of S and G units, which seems to have a similar concentration, having a low concentration of H units. This composition is characteristic of hardwood lignins^52^ and was also indicated by ^31^P NMR results. In addition, a signal related to cinnamyl aldehyde structure (CA) at δ_C_/δ_H_ 125/7.8 was also observed.^48^

**Figure 2:**
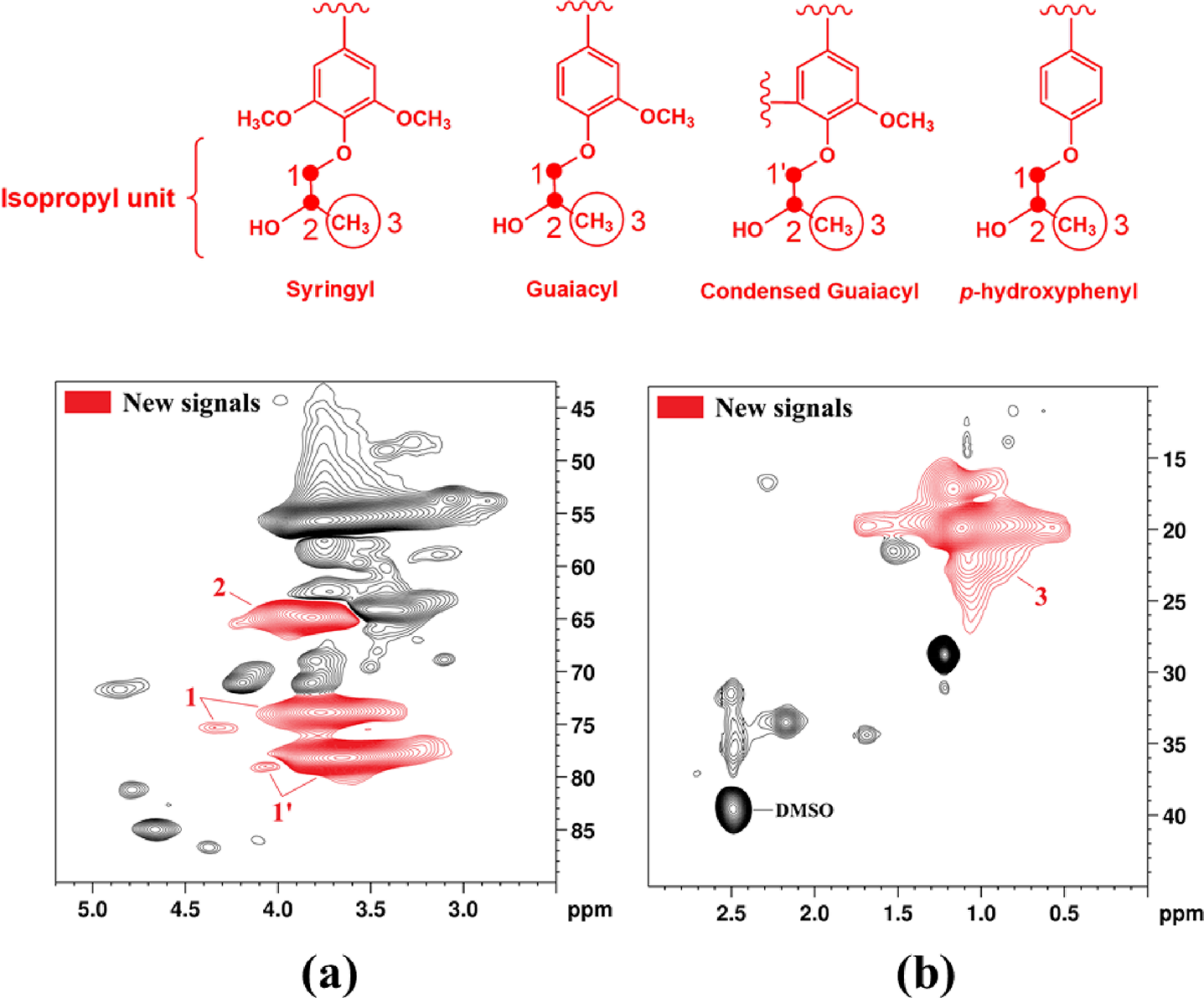
HSQC spectrum of hydroxypropylated lignin (HKL_PO) and assignments of new signals compared with the HSQC spectrum of KL: (a) oxygenated aliphatic region and (b) nonoxygenated aliphatic region.

The oxygenated and nonoxygenated aliphatic regions of the HKL_PO HSQC spectrum are presented in Figure 2. For the first time in the literature, 2D HSQC spectra of hydroxypropylated Kraft lignin from the reaction with propylene oxide under mild conditions are reported. Li et al.^53^ also characterized hydroxypropylated lignin using this technique, but the modification was carried out under a high temperature and pressure (150 °C and 10 atm, respectively), and an organosolv lignin was employed. Compared with the HSQC spectrum of KL (Figure 1a,b), new strong signals were observed in both the oxygenated and nonoxygenated aliphatic regions (Figure 2a,b). In the oxygenated aliphatic region, three new signals emerged: δ_C_/δ_H_ 63–67/3.6–4.2, 72–76/3.3–4.1, and 76–80/3.1–4.0. Those signals are associated to −CH and −CH_2_ linked by ether bonds in the hydroxypropyl units attached to the lignin structure after hydroxypropylation, which are illustrated in Figure 2.^54^ Additionally, a broad signal appeared at δ_C_/δ_H_ 15–27/1.8–0.4 in the nonoxygenated aliphatic region, indicating the addition of new methyl groups from the hydroxypropyl moieties.^53^ In accordance with ^31^P NMR analysis (Table 1), these results confirm the chemical modification of KL by hydroxypropylation.

### Characterization of the Polyurethane Prepolymers

#### Fourier-Transform Infrared Spectroscopy (FTIR)

**Figure 3:**
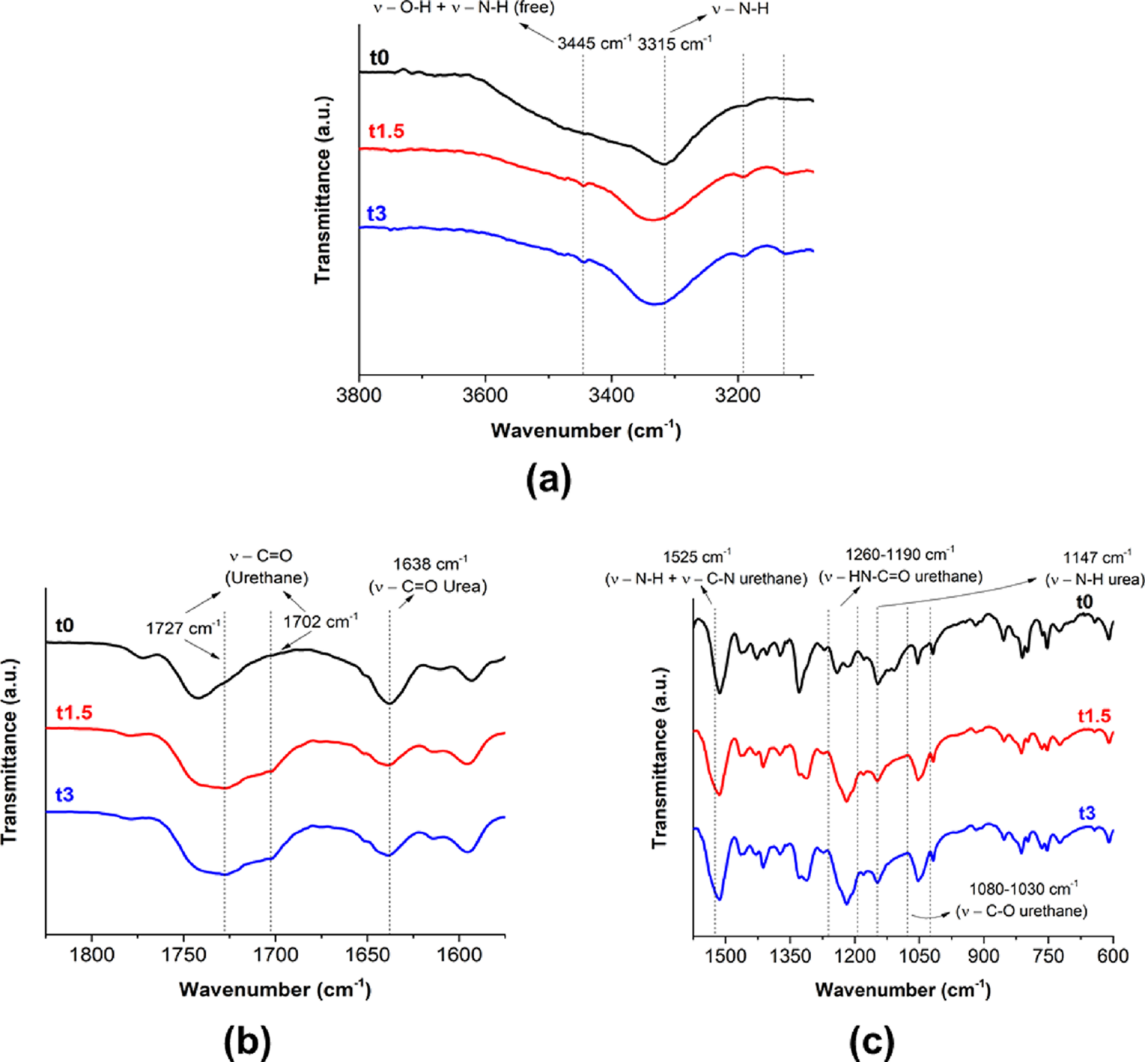
FTIR-ATR spectra of the BPUP_30HKL_PO sample for different prepolymerization times: (a) 3800 to 3100, (b) 1825 to 1575, and (c) 1575 to 600 cm^–1^.

**Scheme 1:**
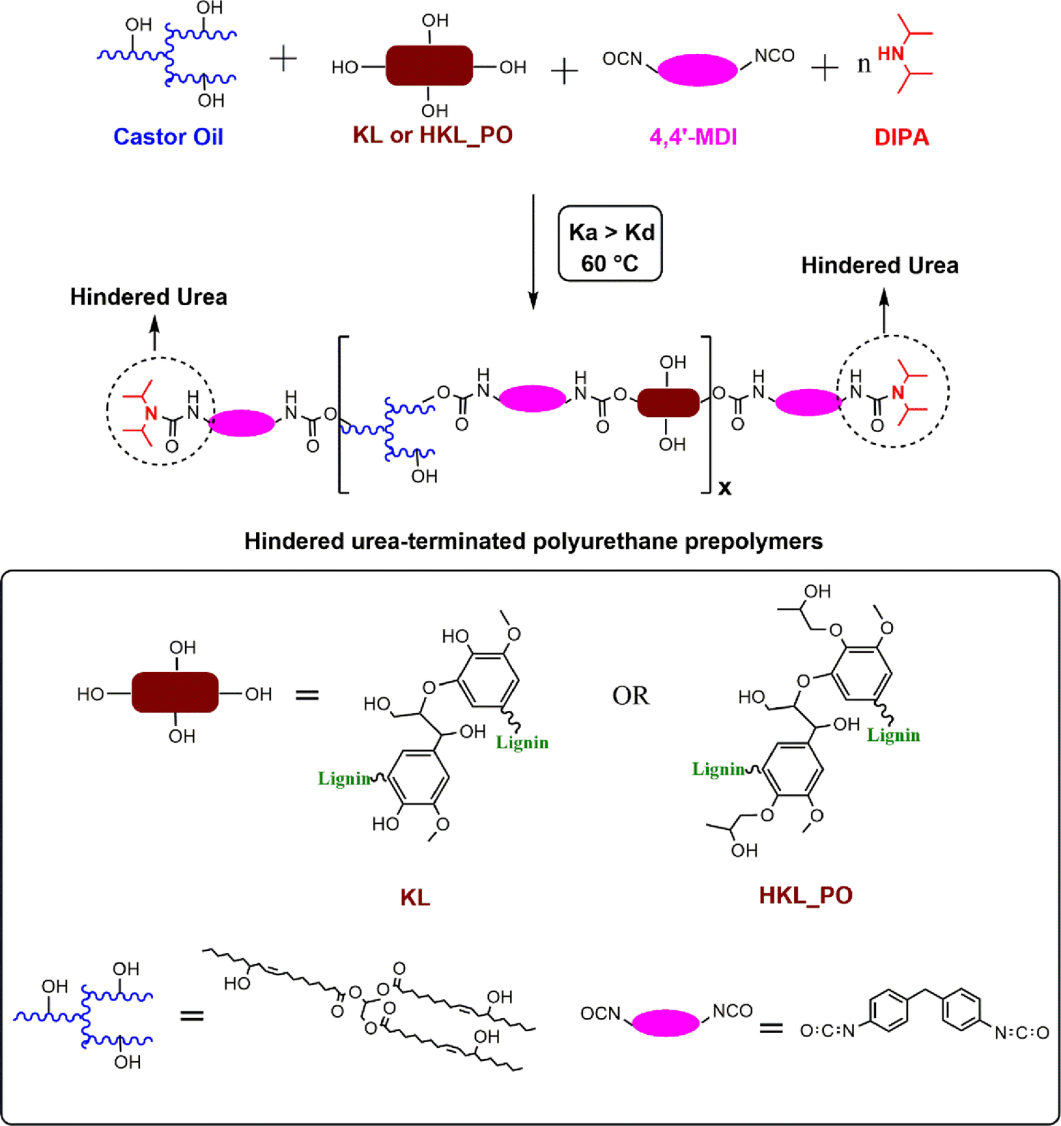
Prepolymerization Based on the Dynamic Urea Bond-Mediated Polymerization (DUBMP) for Obtaining Hindered Urea-Terminated Polyurethane Prepolymers from KL or HKL_PO

The prepolymerization of BPUP_30KL and BPUP_30HKL_PO, which is schematized in Scheme 1, was followed by FTIR-ATR spectroscopy. The BPUP_30KL spectra for different prepolymerization times (0, 1.5, and 3 h) are shown in Figure S3 (Supporting Information), whereas the spectra of BPUP_30HKL_PO for the same prepolymerization times are displayed in Figure 3. All spectra were normalized with respect to the peak at 1515 cm^–1^ (C=C aromatic bond vibration). In order to improve the analysis, the BPUP_30HKL_PO spectra were divided into three specific regions. A broad band between 3700 and 3200 cm^–1^, which is associated with the stretching mode of N–H bonds of urethane and urea and hydroxyl groups,^55^ was observed at the beginning (*t*_0_) of prepolymerization (Figure 3a). This band revealed the instantaneous formation of urethane and mainly hindered urea groups that is proven by the presence of the shoulder at 1727 cm^–1^ corresponding to the stretching of the urethane carbonyl group and the band centered at 1638 cm^–1^ assigned to the stretching of urea carbonyl (Figure 3b).^56^ Some evidence for the formation of urethane linkages during the prepolymerization was verified: a decrease of the O–H stretching band intensity (between 3600 and 3400 cm^–1^), indicating the consumption of hydroxyls through the reaction with isocyanates; the band related to the N–H stretching centered at 3315 cm^–1^ that is blueshifted (N–H linkages of urethanes absorb at a higher wavenumber than urea ones)^57^ and its intensity increased; the width increase of the band between 1760 and 1670 cm^–1^, which is associated with the stretching mode of carbonyls, and the emergence of a new peak (1703 cm^–1^); and the increase in the intensity of the bands around 1525 cm^–1^, between 1260 and 1190 cm^–1^, and between 1080 and 1030 cm^–1^, assigned to the combination of the stretching of the N–H and C–N bonds,^58^ the stretching of C=O bonds connected to N–H bonds,^59^ and the stretching of C–O bonds,^60^ respectively (Figure 3c). Regarding the hindered urea group, the intensity of the assigned bands at 1638 and 1147 cm^–1^ almost remained constant, revealing the higher stability of this group under the reaction conditions.^61^ Under this condition (60 °C), the association constant between DIPA and isocyanate groups (*K*_a_) is higher than the dissociation constant (*K*_d_), as shown in Scheme 1, and the formed hindered urea groups tend to be chemically quite stable.

**Figure 4:**
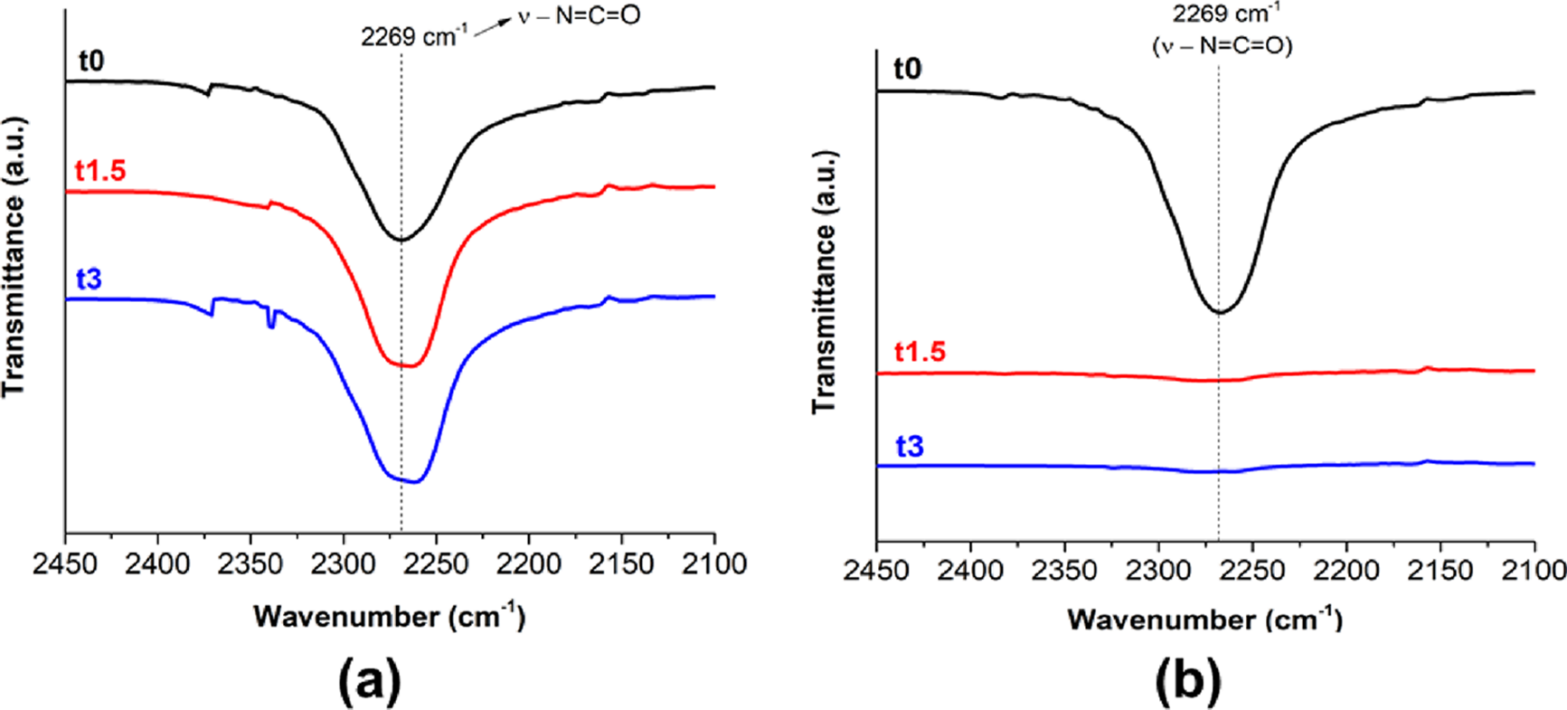
FTIR-ATR spectra of (a) BPUP_30KL for different prepolymerization times and (b) BPUP_30HKL_PO for different prepolymerization times.

With a comparison with the spectra of BPUP_30KL, a similar trend was verified for all spectral regions, except for the isocyanate band region (2450 to 2100 cm^–1^; Figure 4). In this region, in the case of the BPUP_30KL sample, no significant changes during the prepolymerization process were observed for the band associated with isocyanate groups centered at 2269 cm^–1^, indicating that there was no significant consumption of isocyanate groups for the formation of urethane groups after 1.5 h (Figure 4a). This result reveals the lower reactivity of KL against isocyanate under the prepolymerization conditions because the hydroxyl groups of KL are inaccessible, even with a high amount of isocyanate available to react. Therefore, it is plausible to assume that only a small amount of KL hydroxyls reacts to MDI to form urethane linkages. Therefore, most urethane linkages formed during the prepolymerization must be derived from the reaction between castor oil and MDI. Furthermore, the fact that the intensity of the isocyanate band remained constant between 0 and 1.5 h suggested that no urethane linkages were formed during this period of time. However, as pointed out before, evidence verified in other spectral regions confirmed the emergence of such a group throughout the prepolymerization process. Although not favored at 60 °C (*K*_d_ of ∼0.20 at 40 °C),^62^ the hindered urea dissociation occurred to a small degree during the prepolymerization, as indicated by the slight reduction in the intensity of the band centered at 1638 cm^–1^ between 0 and 3 h (Figure S3). This minor process provides free isocyanates and, in follow-up reactions, urethanes to the system. On the other hand, for BPUP_30HKL_PO, after 1.5 h, almost all isocyanate groups had already been consumed (Figure 4b). This result indicates that the HKL_PO has a considerably higher reactivity than KL at the reaction conditions. Therefore, different from BPUP_30KL, in which KL did not participate in the formation of urethane linkages, HKL_PO contributed significantly more to the formation of urethane groups and, consequently, to the synthesis of the blocked PU prepolymer.

#### Temperature-Modulated Optical Refractometry (TMOR)

**Figure 5:**
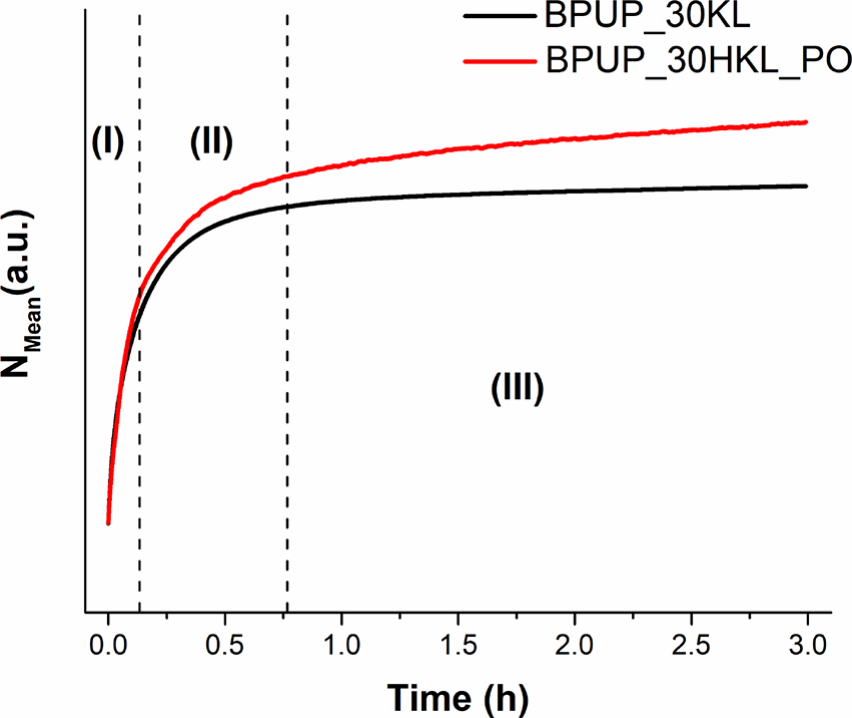
Temporal evolution of the average refractive index (*N*_Mean_) during the polymerization of blocked polyurethane prepolymers at 60 °C.

TMOR analysis was used to monitor the average refractive index (*N*_Mean_) of the blocked PU prepolymers during their prepolymerization. The results are shown in Figure 5. Comparing the curves, a similar behavior of increasing *N*_Mean_ with time, which is associated with formation of urethane linkages during prepolymerization and the consequence densification,^63^ was observed for both samples, albeit with slight differences. Based on the shape of the curves, the prepolymerization process can be divided into three stages (I, II, and III) related to different reaction rates. These stages are indicated by dashed vertical lines in Figure 5. These three stages were already verified by other works and are characteristic of this polymer.^22,33,63^

During the first stage (I), a significant increase in *N*_Mean_ was observed, which is a consequence of a high reaction rate.^63^ In this stage, *N*_Mean_ varied almost linearly with time, and no significant differences were observed between the two systems. Therefore, it is plausible to conclude that this first step is governed by the reaction between castor oil and MDI, as the castor oil shows higher reactivity than both lignins, due to the lower molar mass and more accessible OH groups, and reacts promptly at the beginning of the prepolymerization. After approximately 0.20 h, in stage II, a reduction of the slope of the *N*_Mean_ vs time curves was observed, indicating a significant decrease in the reaction rate of BPUP_30KL and BPUP_30HKL_PO prepolymerization. This behavior is a consequence of two factors: (a) the decrease in reagent concentrations after their consumption and (b) the decrease in molecular mobility due to a molecular weight increase, which slows further diffusion of the reactants.^22,45^ Despite showing similar trends, the system containing HKL_PO displayed higher reaction rates throughout stage II than the one containing KL. This result suggests that the modified lignin has a higher reactivity compared to the unmodified one, which is in agreement with the FTIR analysis (Figure 4).

In stage III, which started at around 0.75 h, *N*_Mean_ reached a plateau shape. This result indicates an ongoing prepolymerization process that proceeds at a very low reaction rate, which slowly approaches zero as a consequence of the high molecular weight that hinders macromolecular mobility in this step.^63,64^ As noted in stage II, also here, BPUP_30HKL_PO showed higher reaction rates than BPUP_30KL, confirming the statement that HKL_PO has a higher reactivity than KL. Even in this critical reaction stage, the prepolymerization continued at a higher rate. Furthermore, in the case of the system containing HKL_PO, the prepolymerization apparently does not cease even after 3 h. Although prepolymerization might still happen to some extent, the employed prepolymerization time was more than sufficient for the formation of the proposed blocked PU prepolymers.

#### Two-Dimensional Nuclear Magnetic Resonance Spectroscopy (HSQC)

**Figure 6:**
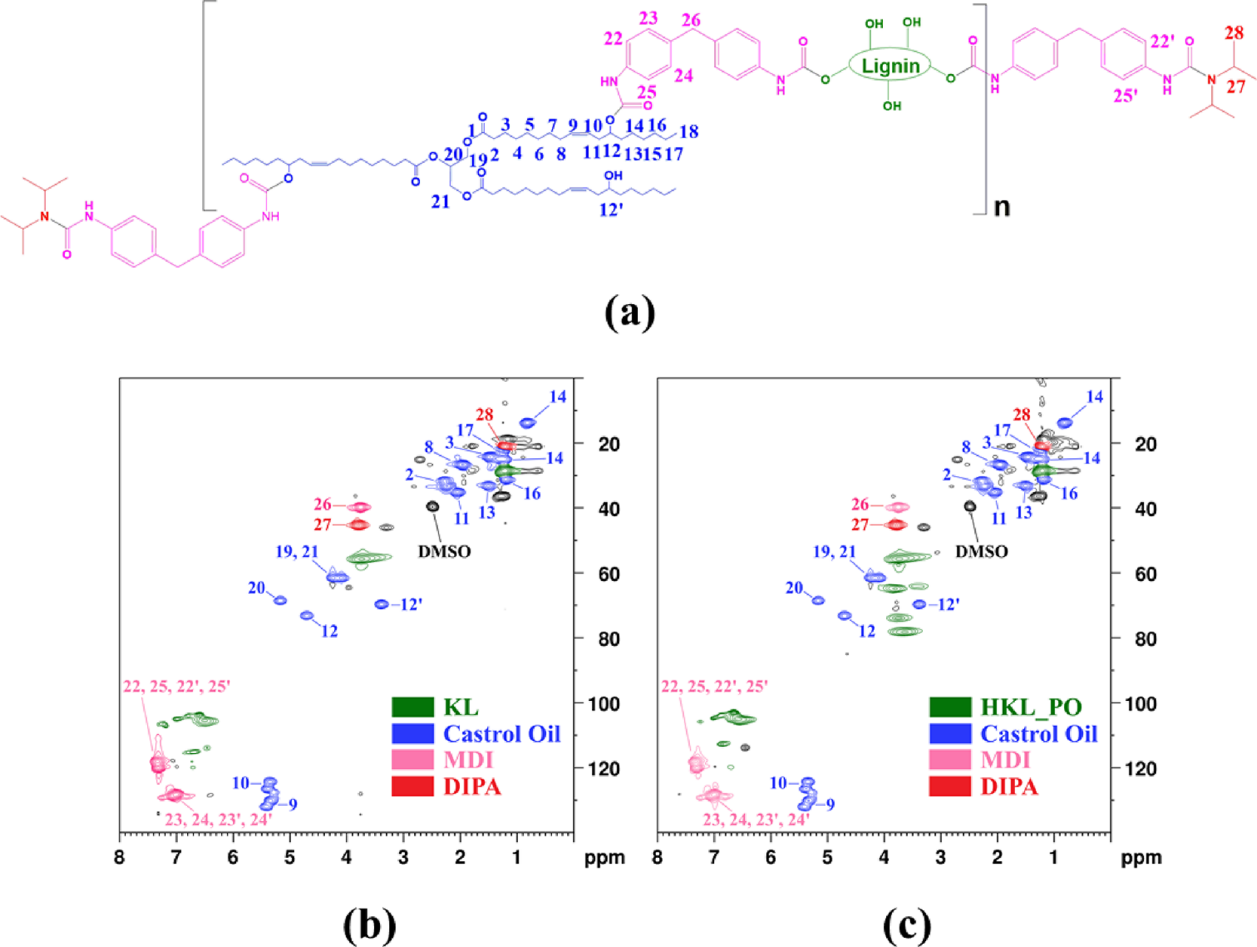
(a) Blocked PU prepolymer structure with some labeled carbons, (b) HSQC spectrum of the BPUP_30KL prepolymer and assignments of some selected signals, and (c) HSQC spectrum of the BPUP_30HKL_PC prepolymer and assignments of some selected signals.

The structure of the prepolymers with labeled hydrogens and carbons and the HSQC spectra of the BPUP_30KL and BPUP_30HKL_PO prepolymers are shown in Figure 6a–c. Signals assigned to all PU precursors (lignin, castor oil, and MDI), as well as the blocking agent (DIPA), were identified for both samples. The C–H cross-peaks associated with castor oil, MDI, and DIPA are highlighted in blue, pink, and red in the spectra, respectively. For castor oil, the following signals were found: δ_C_/δ_H_ C_2_ = 33/2.20, C_3_ = 24/1.50, C_8_ = 29/1.97, C_9_ = 128 to 134/5.20 to 5.50, C_10_ = 123 to 128/5.20 to 5.50, C_11_ = 35/2.05, C_12_ = 74/4.80, C_12’_ = 71/3.40, C_13_ = 33/1.40, C_14_ = 24/1.15, C_16_ = 31/1.10, C_17_ = 22.5/1.20, C_18_ = 14.5/0.86, C_19,21_ = 57 to 63/3.90 to 4.30, and C_20_ = 68/5.15. In the case of MDI, the signal was found at around δ_C_/δ_H_ C_22,25,22′,25′_ = 113 to 127/7.15 to 7.50, C_23,24,23′,24′_ = 127 to 131/6.65 to 7.30, and C_26_ = 40/3.80. Finally, for DIPA, the signals verified were δ_C_/δ_H_ C_27_ = 46/3.80 and C_28_ = 21/1.25. The signals associated with both lignins were circled in green. In addition to the NMR spectra simulation tool (nmrdb.org), some studies were consulted for the signal assignment.^65−67^

The HSQC NMR results confirmed that the PU prepolymers were formed from all starting materials, even for BPUP_30KL, in which KL contributed little to the formation of urethane linkages, as revealed by the FTIR analyses. Furthermore, the signals assigned to DIPA and the higher number of signals associated to aromatic carbons of MDI are evidence of the presence of hindered urea groups at the ends of both prepolymer chains, which was also confirmed by FTIR analysis. Last but not the least, the signal observed at δ_C_/δ_H_ 71/3.40 ppm reveals the existence of some residual hydroxyls from castor oil in the prepolymers, as expected.

### Single-Lap Shear Test

**Scheme 2:**
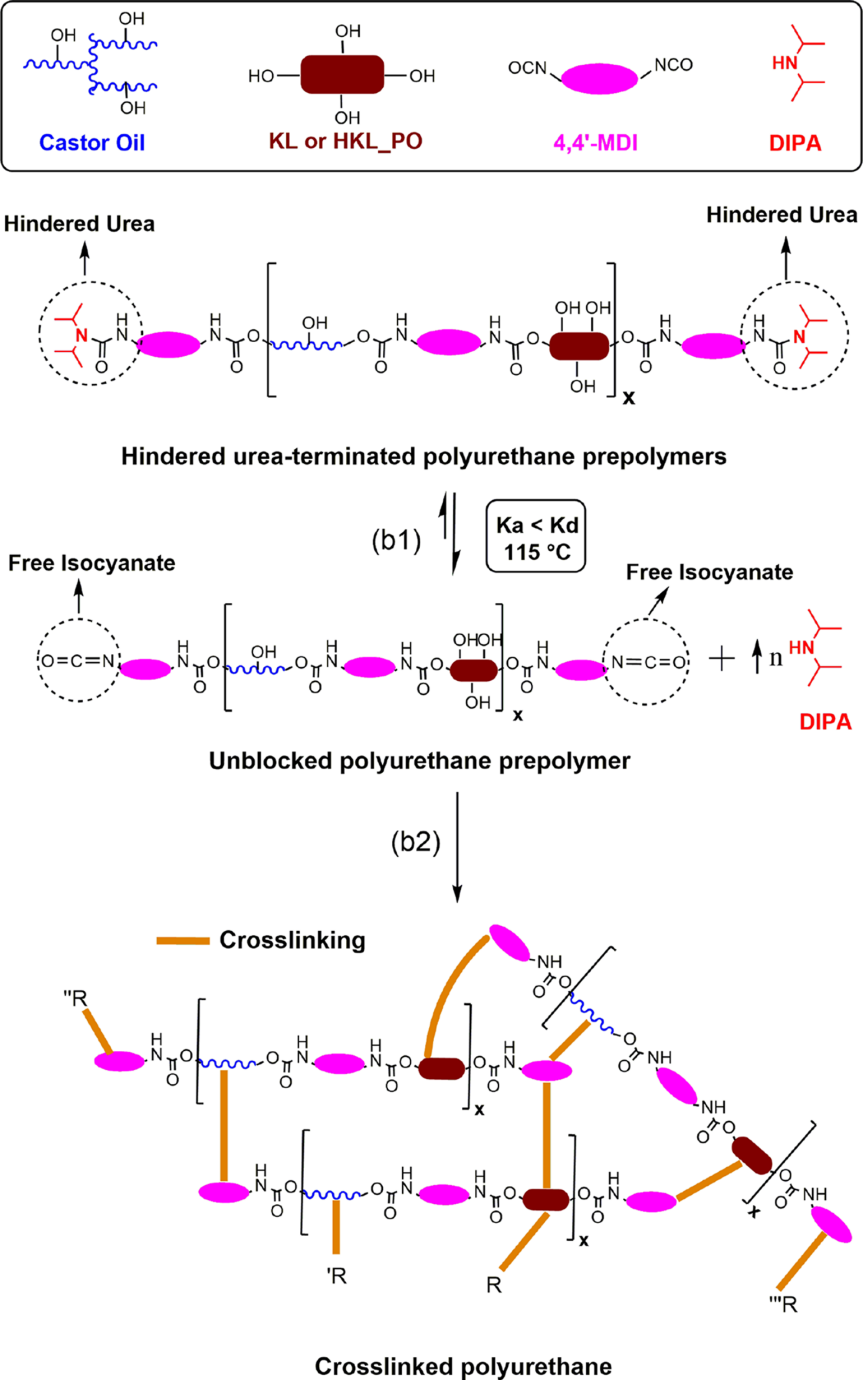
Curing Process Mechanism of LPUs Based on Isocyanate Groups Unblocking

The possibility of applying the synthesized LPUs as adhesives was evaluated by single-lap shear tests. Before showing the test results, it is worth describing the curing reaction mechanism of the LPUs. The curing reaction, which is shown in Scheme 2, is based on isocyanate groups unblocking. At 115 °C, *K*_d_ becomes higher than *K*_a_.^40^ Consequently, the hindered ureas dissociate into isocyanate and DIPA again (reaction b1 of Scheme 2). Due to its low boiling temperature (84 °C), DIPA is then evaporated, and the isocyanate groups, previously blocked, are free to react with residual hydroxyls and also with the surface of substrates in the case of adhesive joints, thus promoting crosslinking of prepolymers (reaction b2 of Scheme 2).

**Figure 7:**
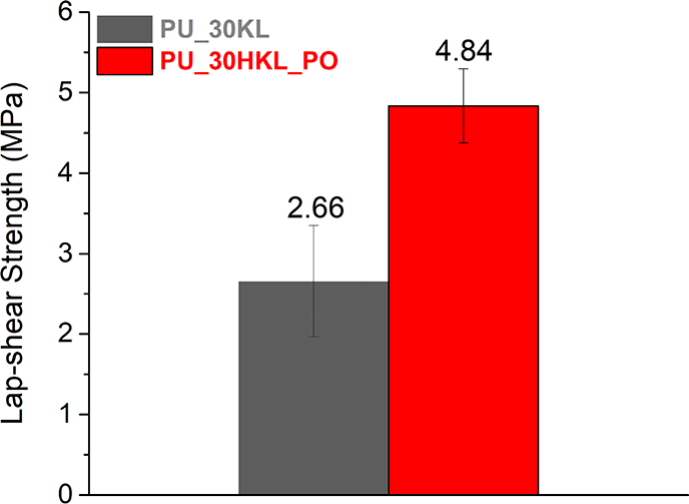
Single-lap shear test average results for synthesized LPUs in steel substrates.

The average lap-shear strengths of the adhesive systems based on BPUP_30KL and BPUP_30HKL_PO prepolymers (PU_30KL and PU_30HKL_PO, respectively) are displayed in Figure 7. Both adhesive systems exhibited predominantly cohesive failure. Comparing the results, PU_30HKL_PO showed a lap-shear strength 82% higher than PU_30KL. As cohesive failures were observed for both systems, the best performance of PU_30HKL_PO is probably related to a higher crosslinking degree. This result is supported by FTIR and TMOR analyses, which indicated that HKL_PO has a higher reactivity than KL. Furthermore, the absolute values of lap-shear strength were similar to those reported in the literature for steel substrates,^22,68^ evidencing the feasibility of using the PU prepolymers as thermoactivated adhesives.

## Conclusions

In this work, novel blocked polyurethane prepolymers based on Kraft lignin (KL), hydroxypropylated lignin (HKL_PO), and castor oil were synthesized and characterized in depth with NMR and FTIR spectroscopic techniques, as well as with temperature-modulated optical refractometry (TMOR). Initially, ^31^P NMR and 2D NMR confirmed that the chemical structure of KL was successfully modified via a hydroxypropylation reaction with propylene oxide. The prepolymerization kinetics studied by FTIR confirmed the formation of hindered urea-terminated polyurethane prepolymers. Furthermore, the FTIR and TMOR results revealed the positive effect of hydroxypropylation on the reactivity of KL. TMOR analysis also showed that the prepolymerization reached a suitable degree after 3 h. The HSQC spectra also confirmed the synthesis of hindered urea-terminated polyurethane prepolymers from both lignin samples and revealed that the prepolymers are made up of all the precursors, including KL. The results proved that the blocked isocyanate approach is feasible for lignin-based polyurethane synthesis, thus contributing to the development of new synthetic strategies for the preparation of these materials. Lastly, the applicability of the prepolymers as adhesives was proven by single lap-shear tests in steel substrates. The adhesive based on the HKL_PO prepolymer exhibited the best performance, revealing the positive effect of hydroxypropylation on the practical adhesion of the systems. Then, the synthesized prepolymers can be applied as monocomponent heat-activated adhesives.

## Supporting Information Available

Brief theoretical background of the TMOR technique, ^31^P NMR spectra for all lignin samples, and FTIR-ATR spectra for the BPUP_30KL sample (PDF)
