# Supplementary material for: Lignin-Based Polyurethanes from the Blocked Isocyanate Approach: Synthesis and Characterization
Source: ACS Omega. 2023 Jul 18;8(30):27621–33. doi: 10.1021/acsomega.3c03422 (PMC10398858; doi:10.1021/acsomega.3c03422)
Supplement: Supplementary file 1 — ao3c03422_si_001.pdf [file ao3c03422_si_001.pdf]

Lignin-based Polyurethanes from the Blocked Isocyanate Approach: Synthesis and Characterization

Leonardo D. Antonino[1], *Ivan Sumerskii*[2], *Antje Potthast*[2], *Thomas Rosenau*[2], *Maria Isabel Felisberti*[3], *Demetrio J. dos Santos*[1,4,*]

[1]Nanoscience and Advanced Materials Graduate Program (PPG-nano), Federal University of ABC (UFABC), Santo André 09210-580, Brazil.

[2]University of Natural Resources and Life Sciences Vienna (BOKU), Department of Chemistry, Division of Chemistry of Renewable Resources, Konrad-Lorenz-Strasse 24, 3430, Tulln, Austria.

[3]Institute of Chemistry, University of Campinas (UNICAMP), P.O. Box 6154, 13083-970 Campinas, SP, Brazil.

[4]Center of Engineering, Modeling and Applied Social Sciences, Federal University of ABC (UFABC), Santo Andre 09210-580, Brazil.

*Correspondence: demetrio.santos@ufabc.edu.br

KEYWORDS: lignin; polyurethane; adhesives; hydroxypropylation; blocked isocyanate; blocked agent.

Supplementary Information

Materials and Methods – Temperature modulated optical refractometry (TMOR)

In short, TMOR analysis is based on the acquisition of the refractive index (N) evolution with respect to time and/or temperature changes. For this, sample (solid or liquid) is placed in contact with a high refractive index sapphire prism and the N measurements are carried out in the total internal reflection regime. Once in the equipment cavity, the sample is subject to a temperature program that could be accompanied by a sinusoidal temperature modulation with defined temperature amplitude (0.1–1 °C) and period (20–120 s). At the end of the analysis, a data list is generated that displays the instantaneous refractive index and temperature (N[t] and T, respectively) in addition to the short-time gliding average value of both quantities (T_{Mean} and N_{Mean}, respectively).[44] TMOR can operate in three temperature modes: (i) isothermal, (ii) temperature ramp, (iii) temperature step. In the present work, the isothermal mode was employed to assess the kinetics of the polyurethane prepolymerization. Hence, the following discussion will be based in this mode.

The acquisition of N(t) allows one to access the mass density according to the well-established Lorenz–Lorentz relationship (Equation 1):

$$\frac{N(t)^2-1}{N(t)^2+2} = r.\,\rho\,(t), \qquad (1)$$

in which ρ is the instantaneous density, and r is the specific refractivity. This latter quantity reflects the atomic or molecular polarizability and might be regarded as constant because, in the case of polymers, the optical dipole is weakly dependent on temperature changes. Thus, assuming r and the mass of the sample as constant throughout the measurement, N can relate to the specific volume v (v = 1/ρ) rearranging Equation 1. Thus, estimate specific volume variation with time can be calculated according to Equation 2. Thus, the incremental decrease in volume and the consequent increase in samples' N due to prepolymerization could be followed and might be used as an evidence of prepolymerization reaction (urethane linkage formation) and/or molecular rearrangement.[20,31]

$$v(t) = r.\frac{N(t)^2+2}{N(t)^2-1} \qquad (2)$$

Results and Discussion – ^{31}P Nuclear Magnetic Resonance spectroscopy (^{31}P NMR)

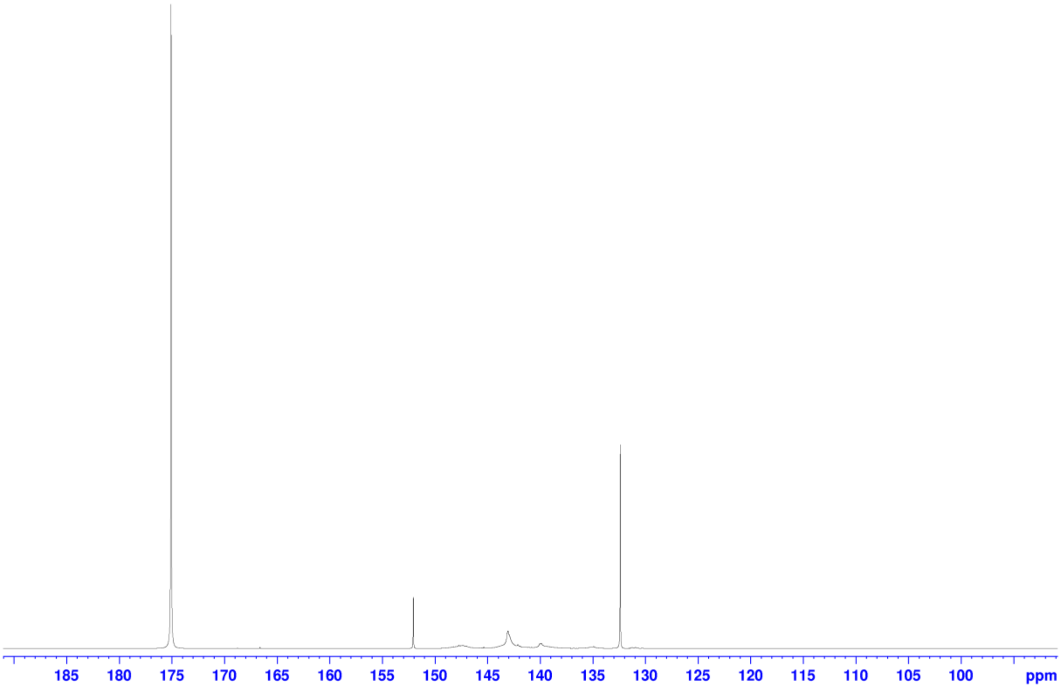

Figure S1. ^{31}P NMR spectrum of KL sample.

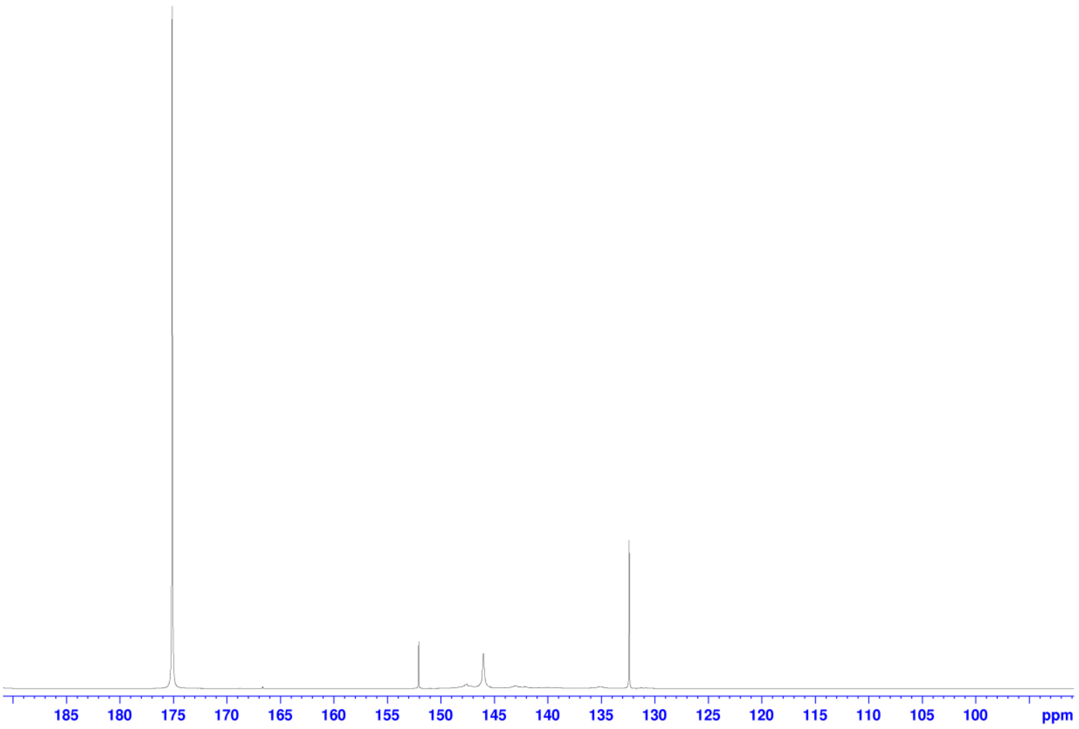

Figure S2. ^{31}P NMR spectrum of HKL_PO sample.

Results and Discussion – Fourier transformed infrared (FTIR)

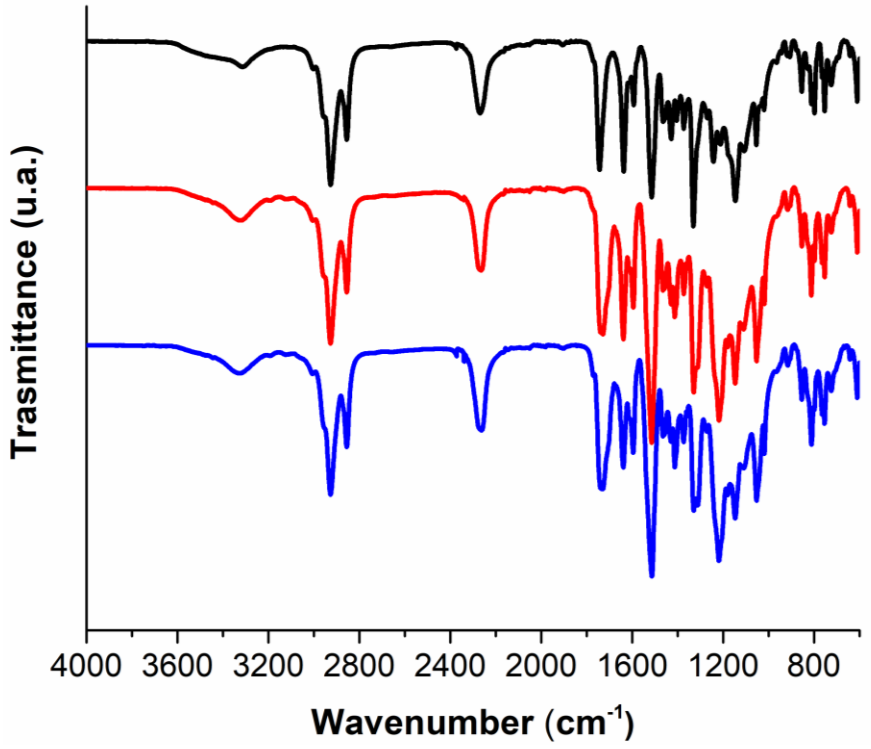

Figure S3. FTIR-ATR of BPUP_30KL sample for different prepolymerization times.
